# Supplementary material for: Selective Sensing of Darolutamide and Thalidomide in Pharmaceutical Preparations and in Spiked Biofluids
Source: Biosensors (Basel). 2022 Nov 11;12(11):1005. doi: 10.3390/bios12111005 (PMC9688659; doi:10.3390/bios12111005)
Supplement: Supplementary file 1 [file biosensors-12-01005-s001.zip › biosensors-2005304-supplementary.pdf]

biosensors

MDPI

Supporting Information

Selective Sensing of Darolutamide and Thalidomide in Pharmaceutical Preparations and in Spiked Biofluids

Wael Talaat [1,*], Abdelbasset A. Farahat [2,3,*] and Reda Mohammed Keshk [4]

[1] Department of Pharmaceutical Analytical Chemistry, Faculty of Pharmacy, Damanhour University, Damanhour 22514, Egypt
[2] Master of Pharmaceutical Sciences Program, California Northstate University, Elk Grove, CA 95757, USA
[3] Department of Pharmaceutical Organic Chemistry, Faculty of Pharmacy, Mansoura University, Mansoura 35516, Egypt
[4] Department of Chemistry, Faculty of Science, Damanhour University, Damanhour 22511, Egypt
* Correspondence: waeltalaat@pharm.dmu.edu.eg (W.T.); abdelbasset.farahat@cnsu.edu (A.A.F.)

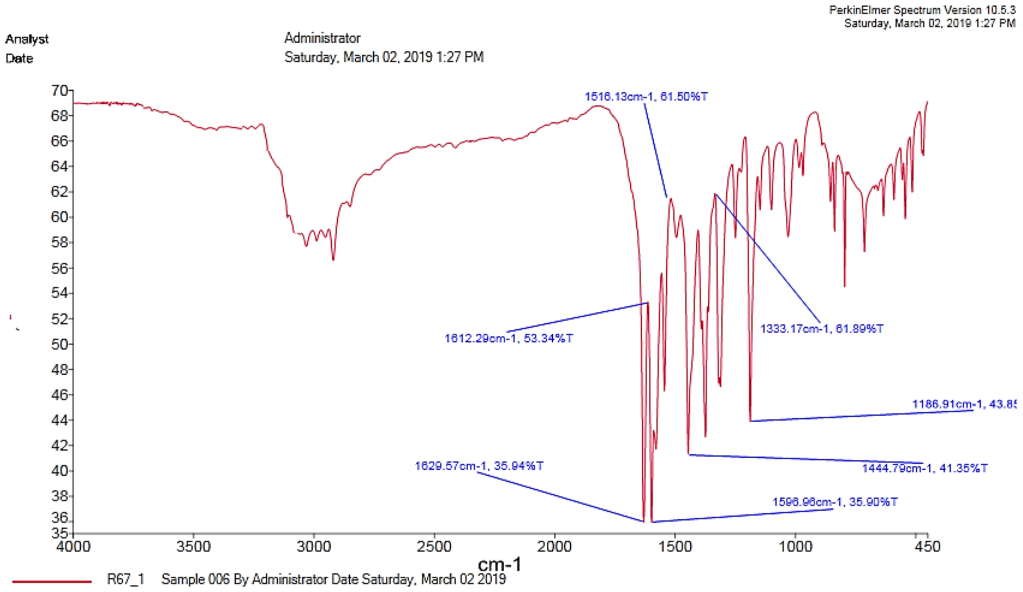

Source Spectra Results	
Spectrum Name	Number Of Peaks
R67_1	7

Figure S1. IR spectra of compound 2 from method A.

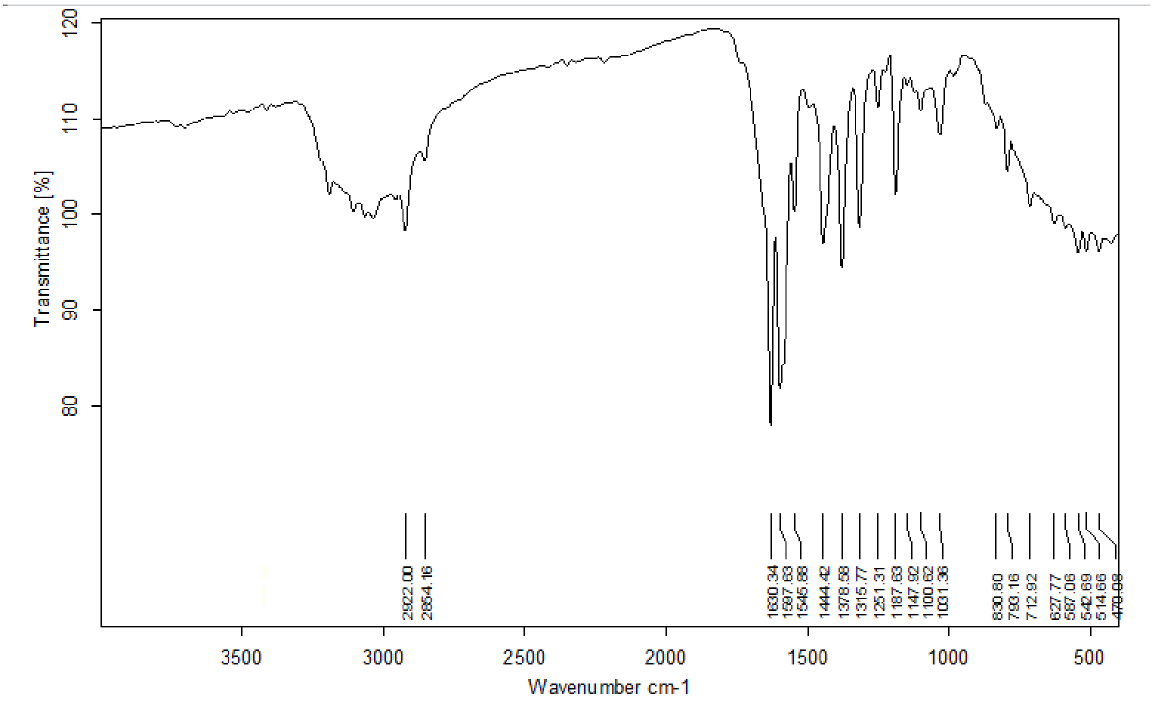

Figure S2. IR spectra of compound 2 from method B.

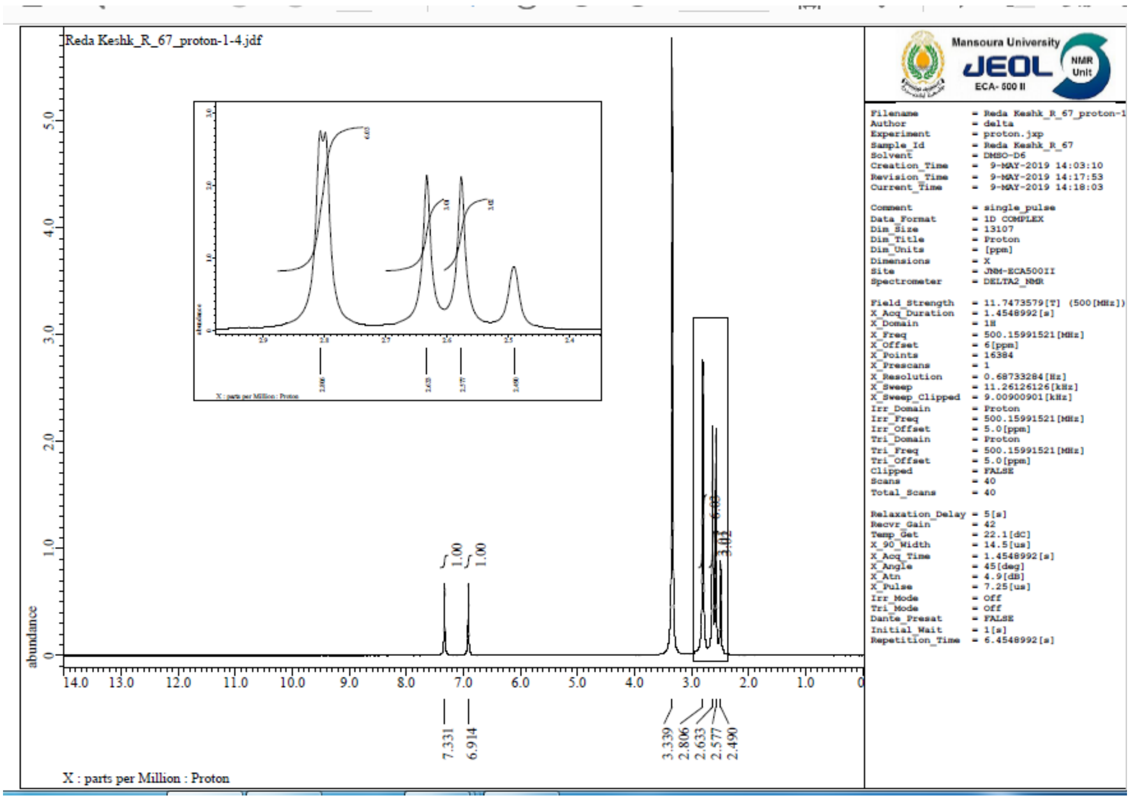

Figure S3. ^{1}H NMR spectra of compound 2 from method A.

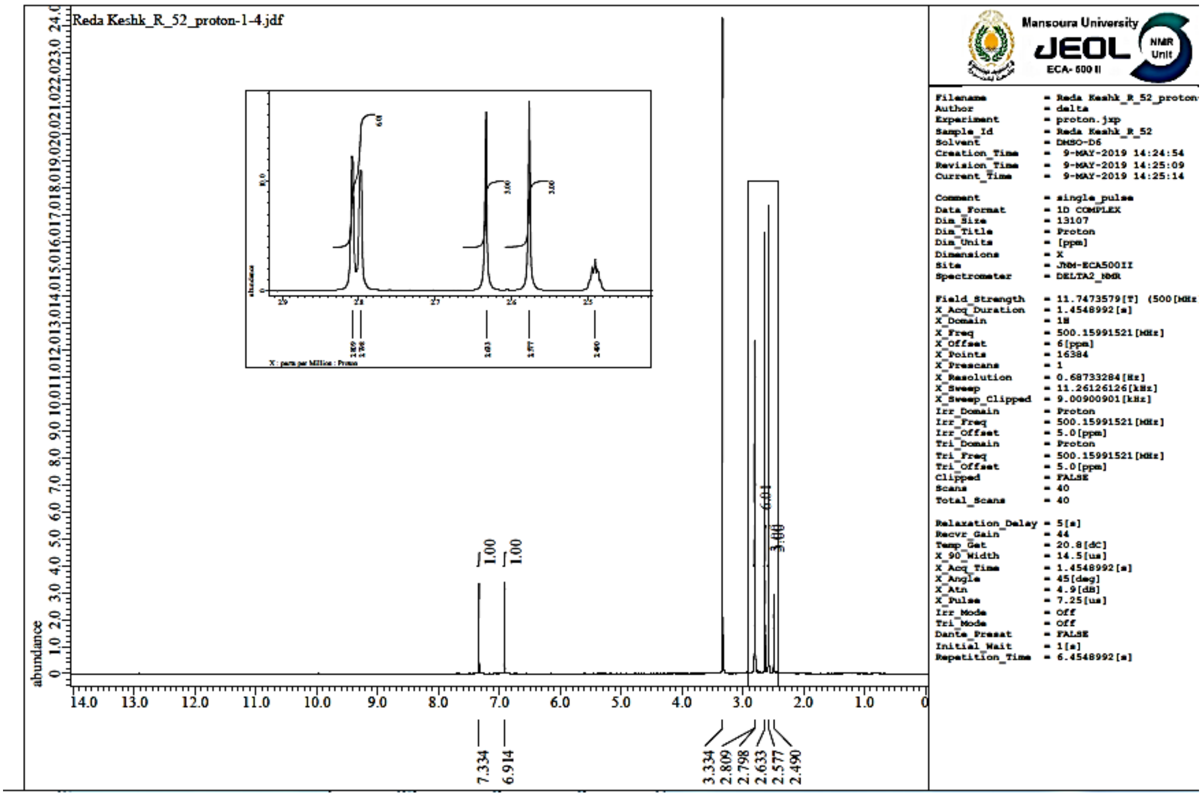

Figure S4. ^{1}H NMR spectra of compound 2 from method B.

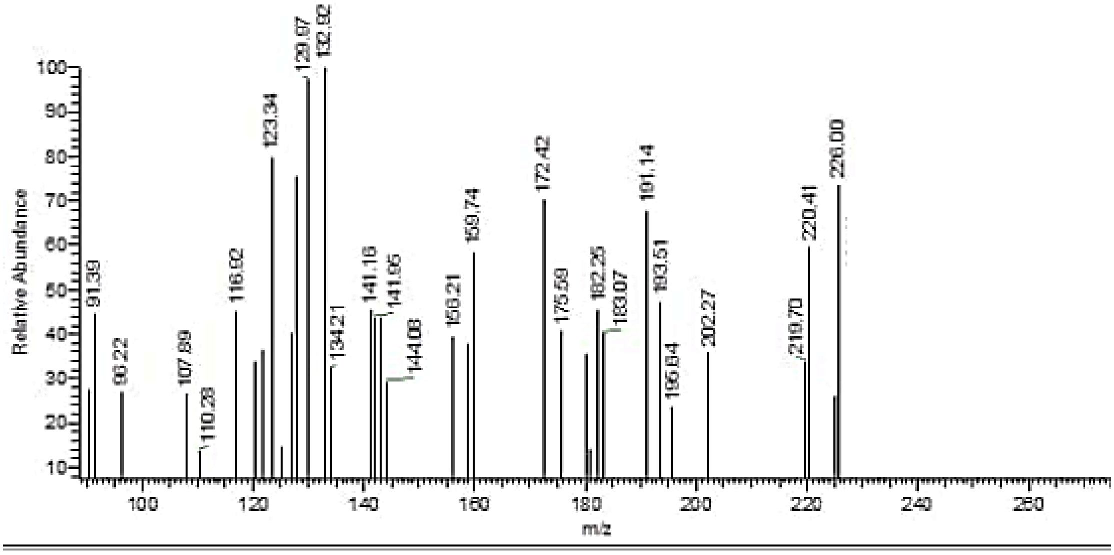

Figure S5. Mass spectrum of compound 2.

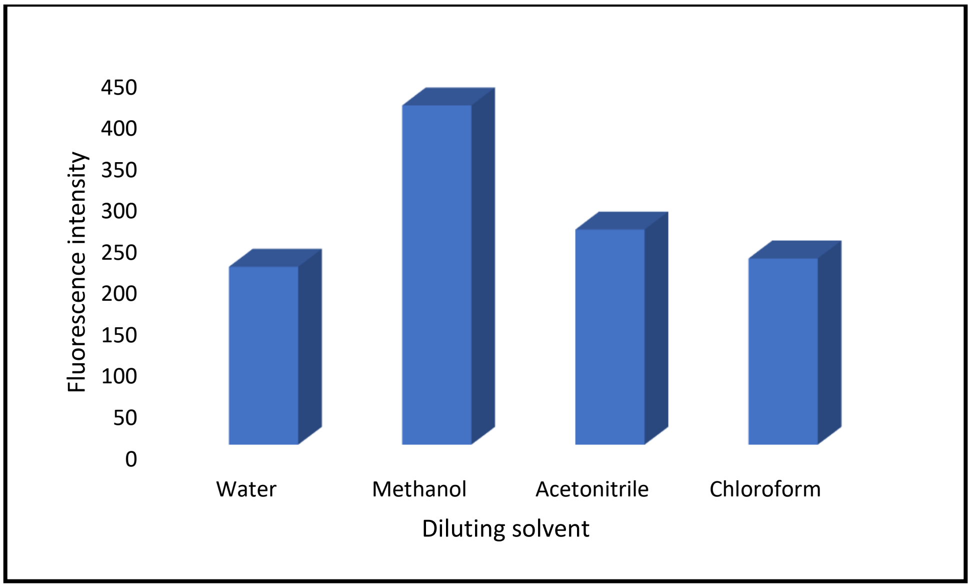

Figure S6. The effect of different diluting solvents on the fluorescence intensity of pyridopyrazolopyrimidine 2.

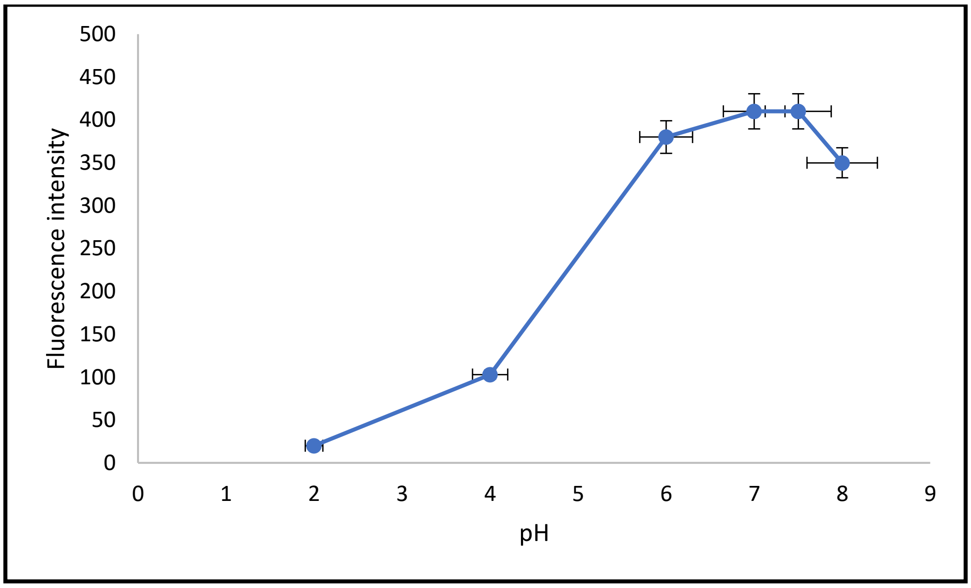

Figure S7. The effect of different pH values on the fluorescence intensity of pyridopyrazolopyrimidine 2.

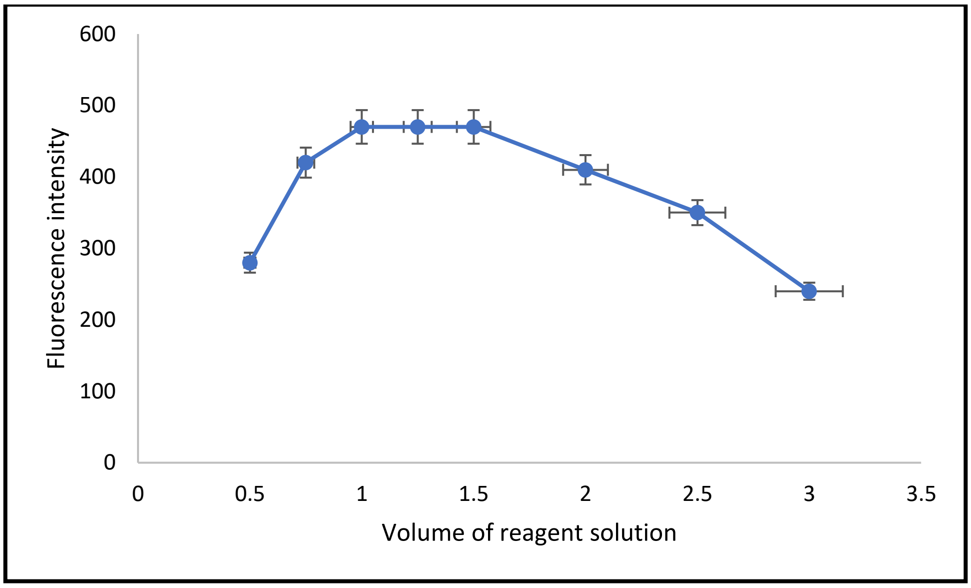

Figure S8. The effect of volume of reagent on the fluorescence intensity of pyridopyrazolopyrimidine 2.

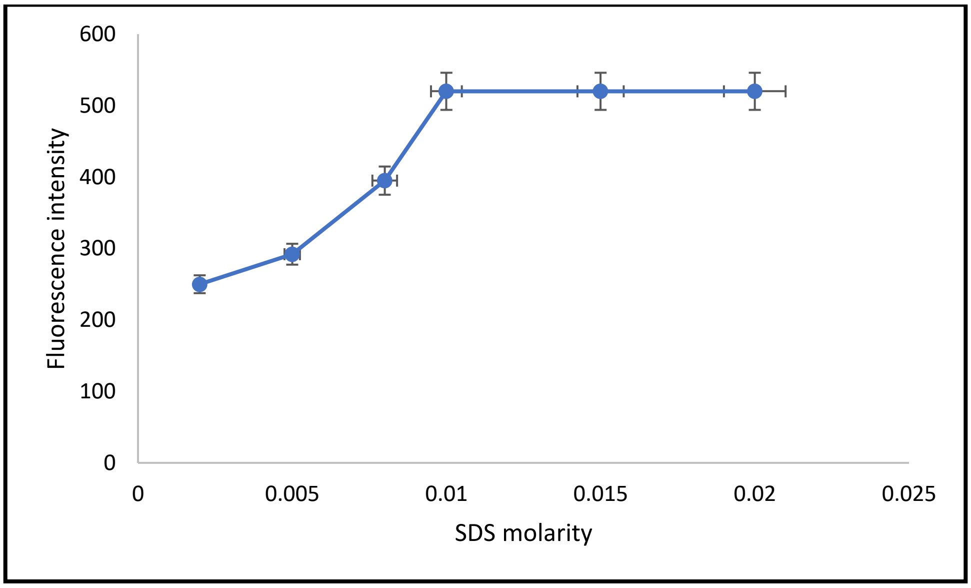

Figure S9. The effect of SDS concentration on the fluorescence intensity of pyridopyrazolopyrimidine 2.

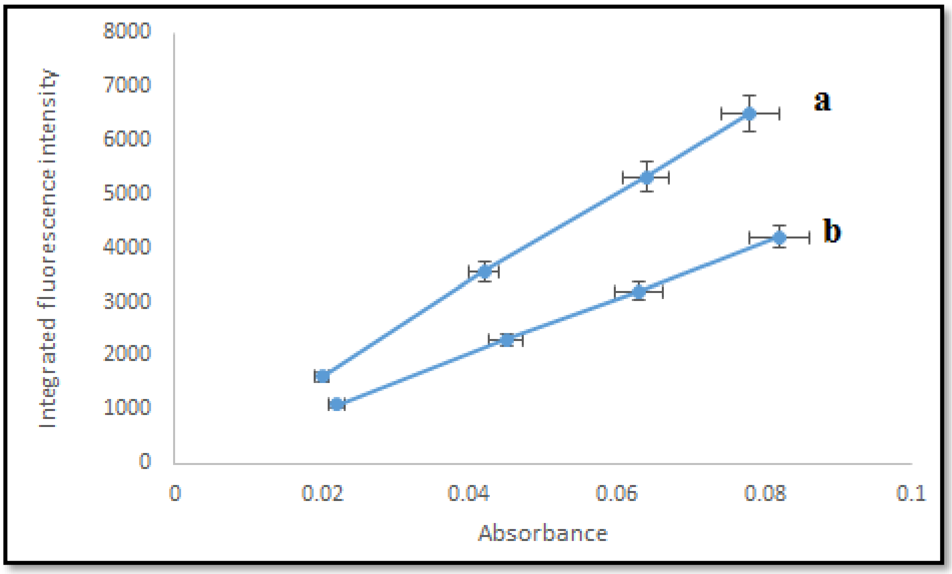

Figure S10. A plot of the integrated fluorescence intensity vs the absorbance of (a) standard quinine sulfate solution, (b) fluorophore solution.

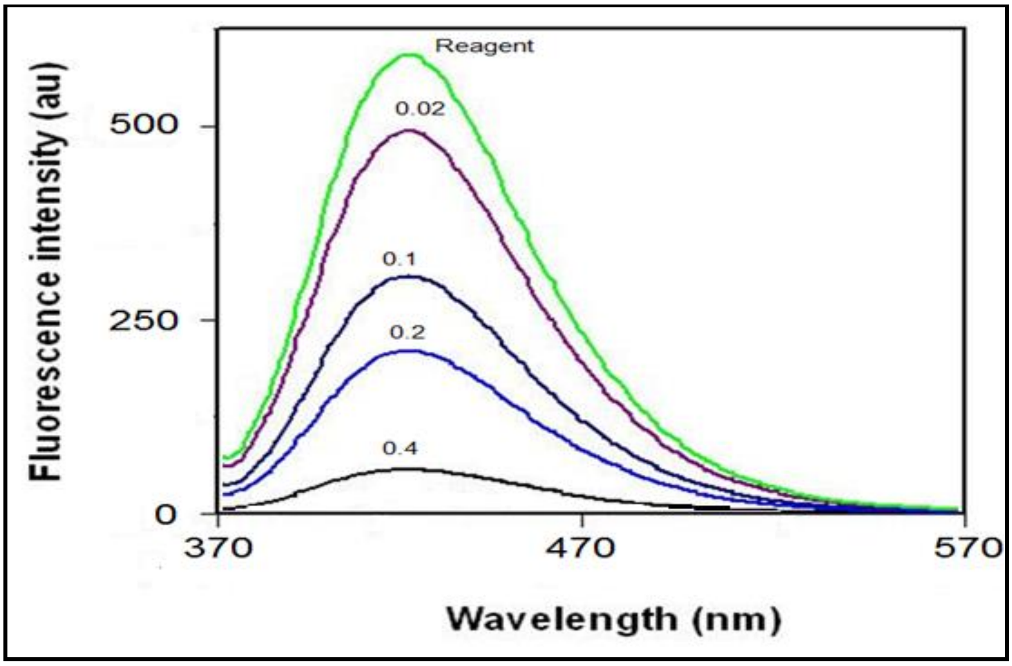

FigureS11: Titrimetric emission spectra with proper concentrations of Darolutamide.

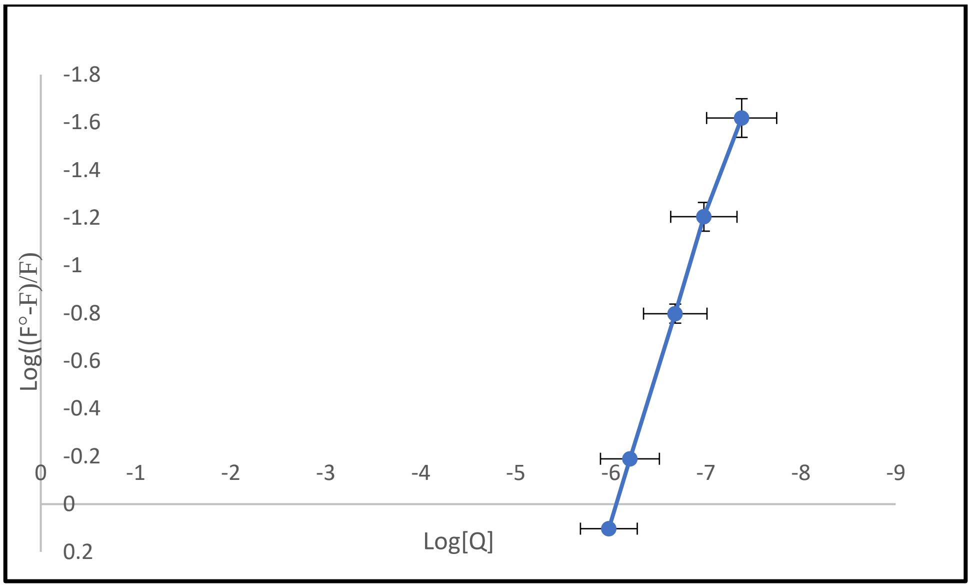

Figure S12. Modified Stern Volmer plot for darolutamide interaction with the reagent.

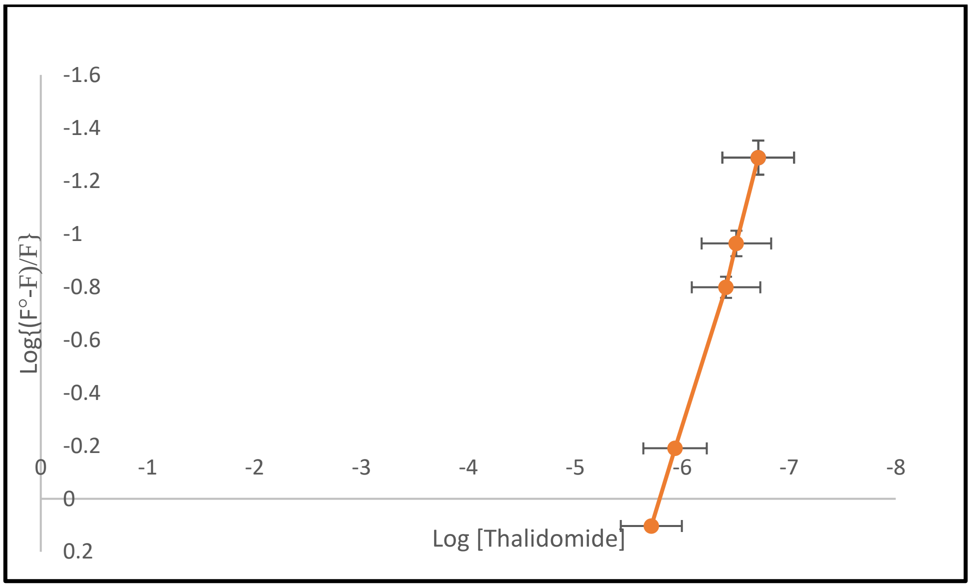

Figure S13. Modified Stern Volmer plot for thalidomde interaction with the reagent.
